# Life on Earth can grow on extraterrestrial organic carbon

**DOI:** 10.1038/s41598-024-54195-6

**Published:** 2024-02-14

**Authors:** Annemiek C. Waajen, Cassio Lima, Royston Goodacre, Charles S. Cockell

**Affiliations:** 1 UK Centre for Astrobiology, University of Edinburgh, Edinburgh, UK; 2 Centre for Metabolomics Research, Department of Biochemistry, Cell and Systems Biology, Institute of Systems, Molecular and Integrative Biology, University of Liverpool, Liverpool, UK

**Keywords:** Carbon cycle, Astrobiology, Meteoritics

## Abstract

The universe is a vast store of organic abiotic carbon that could potentially drive heterotrophy on habitable planets. Meteorites are one of the transporters of this carbon to planetary surfaces. Meteoritic material was accumulating on early Earth when life emerged and proliferated. Yet it is not known if this organic carbon from space was accessible to life. In this research, an anaerobic microbial community was grown with the CM2 carbonaceous chondrite Aguas Zarcas as the sole carbon, energy and nutrient source. Using a reversed ^13^C-stable isotope labelling experiment in combination with optical photothermal infrared (O-PTIR) spectroscopy of single cells, this paper demonstrates the direct transfer of carbon from meteorite into microbial biomass. This implies that meteoritic organics could have been used as a carbon source on early Earth and other habitable planets, and supports the potential for a heterotrophic metabolism in early living systems.

## Introduction

Meteorites heavily bombarded the surface of the early Earth during the origin, early evolution and proliferation of life, potentially providing a source of abiotic organic carbon to sustain early life^1–4^. During the formation of Earth, the hot accretion process would have pyrolysed all existing organic material, meaning that all organics on early Earth must have formed after the planet cooled down, or have been delivered by extraterrestrial sources such as meteorites and micrometeorites^5,6^.

Carbonaceous chondrites, a type of carbon-rich primordial meteorite, have experienced limited alteration by either differentiation or heating in a parent body, and therefore contain organics from the early Solar System^7,8^. The organics in carbonaceous chondrites are therefore representative of the organics that would have been delivered to early Earth. Carbonaceous chondrites contain a wide variety of biologically relevant abiotic organic molecules, such as nucleobases, amino acids and macromolecular organics like polycyclic aromatic hydrocarbons (PAHs)^9–12^. Microbial growth on carbonaceous meteoritic material has previously been shown by Mautner et al.^13–15^. However, these experiments were accomplished under aerobic conditions and used meteoritic extracts derived at high temperatures, which are unlikely to represent conditions on early Earth. While microbial growth under anaerobic conditions on an untreated carbonaceous meteorite was previously shown by Waajen et al.^16^, the direct use of organic carbon in carbonaceous meteorites to synthesise cell biomass has not yet been shown.

Therefore, a fundamental question remains as to whether extraterrestrial organic material could have powered the earliest life on Earth and similar habitable planets. It is hypothesised that the first life on Earth was autotrophic (e.g. Refs.^17,18^), supported by the hypothesis of rapid depletion of available organic molecules for early heterotrophy. The use of constantly replenished meteoritic organics as a substrate for the first organisms was previously dismissed^19^ on the basis that the heterogeneous nature of these organics would have limited their potential as substrates for life, leading to a discounting of a heterotrophic origin of life. In contrast, the hypothesis of a heterotrophic nature of the first living systems has been supported by others^20,21^, who stated that heterotrophic metabolisms of protocells are sustainable, in contrast to early autotrophic metabolisms, which would leak out internally generated metabolites^21^.

Here, we show that meteoritic organic carbon is used by anaerobic microorganisms as the sole carbon source for growth and is incorporated in the microbial proteins, using a single-cell experimental setup combining reverse ^13^C-stable isotope labelling with infrared spectroscopy. This method has enabled tracking the incorporation of the meteoritically-derived carbon into microbial biomass by monitoring molecular vibrations of bacteria at the single-cell level through optical photothermal infrared (O-PTIR) spectroscopy. These results show that organic carbon in space is accessible to current anaerobic life, demonstrating that this organic material would potentially have been available to power the first heterotrophic organisms before the large-scale build-up of biomass on the planet. These findings show that the vast quantities of organic carbon synthesised in early protoplanetary discs can be one source of energy to support early planetary biospheres. Moreover, the biological availability of abiotic extraterrestrial organics is promising for in situ resource utilisation during human space exploration.

## Results

### Microbial use of extraterrestrial organic carbon

**Figure 1:**
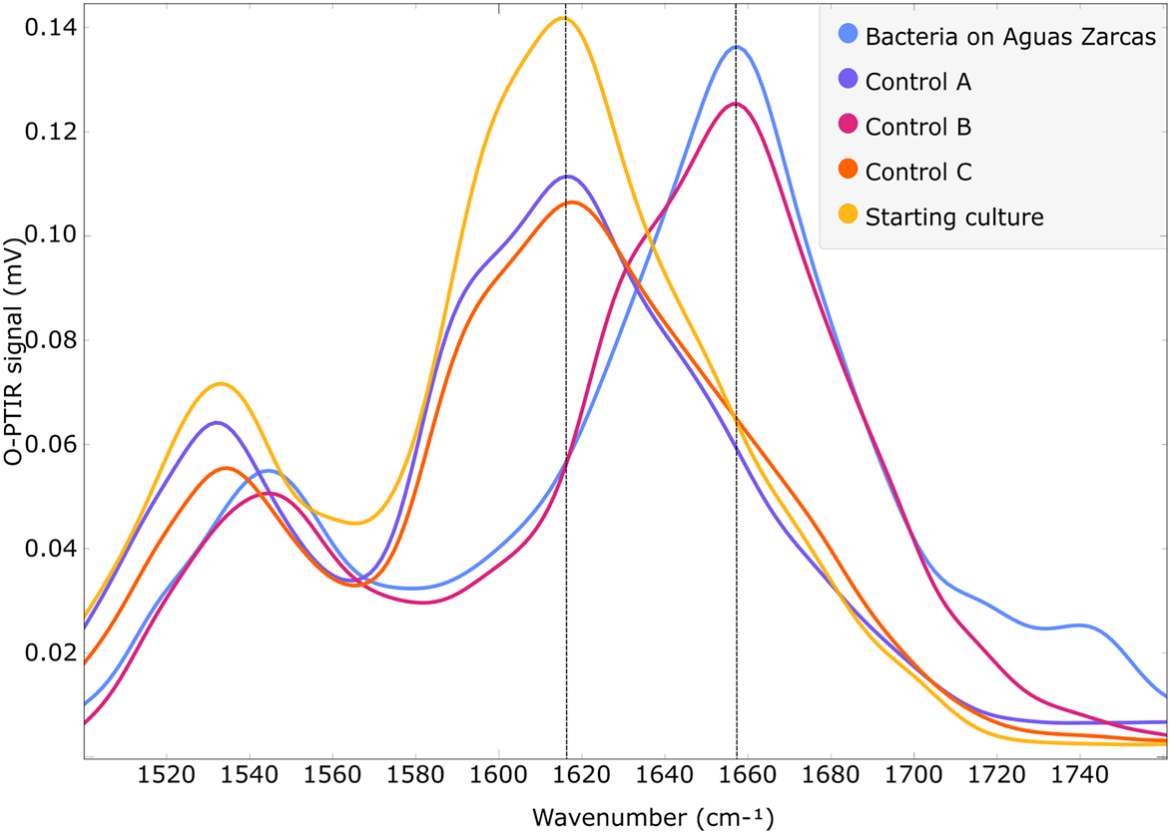
Microbial uptake of organic material from the carbonaceous chondrite Aguas Zarcas. Optical photothermal infrared (O-PTIR) spectroscopy spectra of biological samples with the amide I peaks indicated by dashed lines. The bacteria in the starting culture, Control A (containing ^13^C-labelled sodium acetate) and C (containing no carbon source) have an amide I peak associated with ^13^C (the carbonyl vibrations are at 1616 cm^−1^). Bacteria on Aguas Zarcas and in Control B (non-labelled sodium acetate) have an amide I peak associated with ^12^C (carbonyl vibrations are at 1657 cm^−1^).

**Figure 2:**
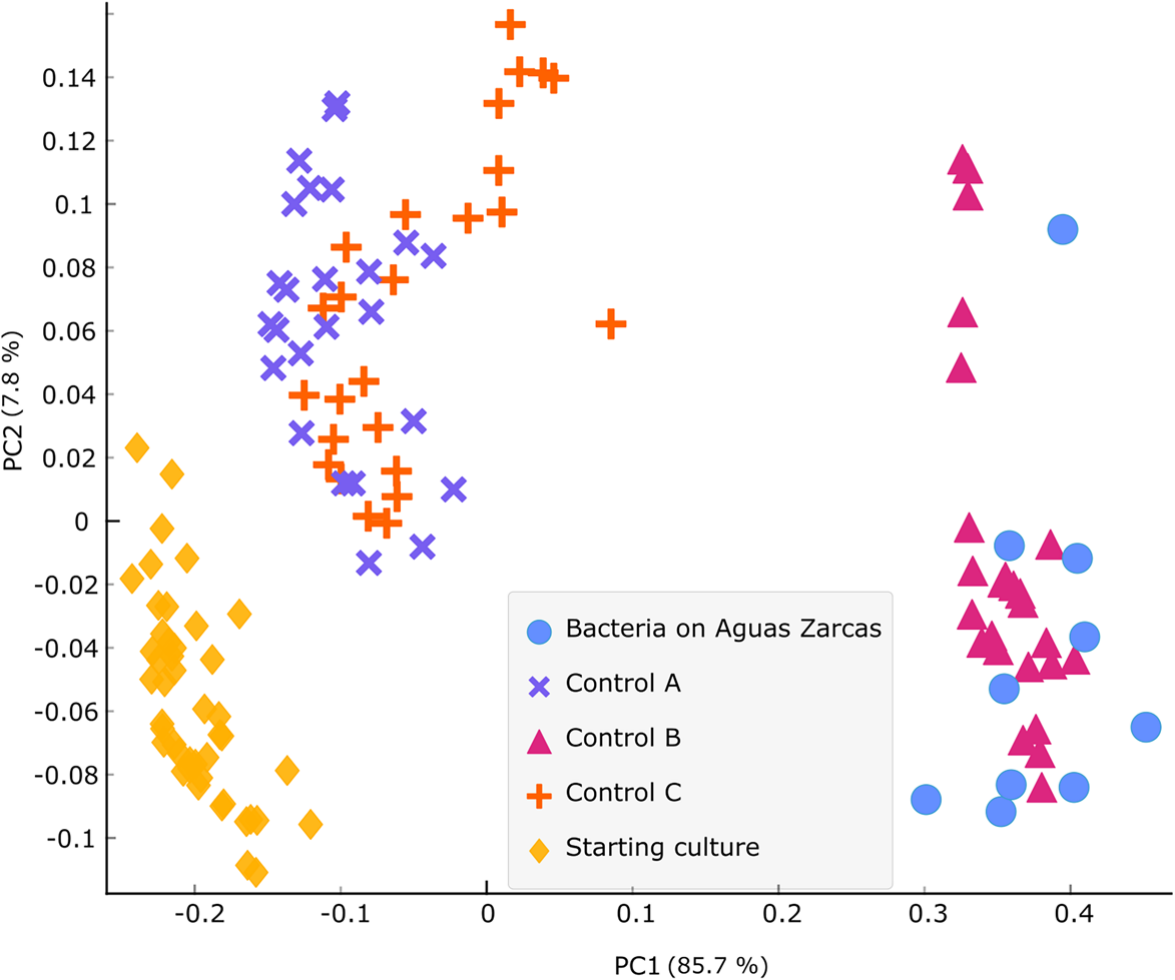
Bacterial differentiation based on the stable isotope of the carbon source. Principal component analysis (PCA) of optical photothermal infrared (O-PTIR) spectroscopy spectra from 1500 to 1760 cm^−1^ of biological samples. Principal component 1 (PC1) separates bacteria grown on a ^12^C carbon source (Bacteria on Aguas Zarcas and Control B) from bacteria grown on a ^13^C carbon source or without an external carbon source (Starting culture, Control A and Control C). See PCA loading plots in Supplementary Fig. S1.

The transfer of the isotopic signature from carbon within the meteorite into an anaerobic microbial community was observed. O-PTIR spectroscopy results showed that microorganisms can use extraterrestrial organic carbon as the carbon source and incorporate it into their proteins. Figure 1 shows the spectral region from 1500 to 1760 cm^−1^, which illustrates vibrational modes associated with proteins (amide I and II regions, 1500–1700 cm^−1^) and lipids (1720–1800 cm^−1^). Peaks between 1580 and 1700 cm^−1^ are associated with the amide I vibrational mode from bacteria grown on ^12^C- and ^13^C-containing carbon sources. Bacteria that are ^13^C-labelled in the starting culture (from growth on ^13^C-labelled sodium acetate) exhibit an amide I peak at 1616 cm^−1^, which corresponds to amide I vibrations where the carbonyl (C=O) is labelled with ^13^C^22^. After transferring the labelled bacteria to microcosms with Aguas Zarcas, containing 1.77 + 0.03% carbon with a δ^13^C value of -0.91 ± 0.25 ‰, a shift was observed in the amide I peak position from 1616 (^13^C) to 1657 cm^−1^ (^12^C). The same ^12^C carbonyl peak was observed in Control B, in which the ^13^C-labelled bacteria from the starting culture were transferred to microcosms containing ^12^C-sodium acetate as the sole carbon supply. When the starting culture was transferred to microcosms containing ^13^C-labelled sodium acetate (Control A), there was no shift in the amide I peak, which remained corresponding to ^13^C (1616 cm^−1^). In Control C, where the starting culture was transferred to microcosms containing no carbon source, the bacteria retained an amide I peak corresponding to ^13^C (1616 cm^−1^). The principal component analysis (PCA) scores plot (Fig. 2) shows a clear separation between bacteria grown in ^12^C-media (on Aguas Zarcas and Control B) and ^13^C-media (starting culture, Control A and C) along the principal component 1 (PC1) axis. By evaluating the PCA loadings (Supplementary Fig. S1), it was concluded that this PC1 separation pattern is mainly based on the amide I peak. Fourier transform infrared (FT-IR) spectroscopy was used to analyse non-biological samples. No amide I peak was observed in the meteoritic material nor the minimal medium in Control A and B (Supplementary Fig. S2). Infrared spectroscopy requires drying the samples before analysis due to the strong contribution of water in the infrared spectrum, therefore spectral data from non-biological Control C showed no vibrational modes (data not shown) as this sample contained solely water.

### Microbial growth on extraterrestrial organic carbon

Bacterial growth of the community was observed in microcosms with Aguas Zarcas, as well as the three controls, although growth was slower in the Aguas Zarcas-containing samples (Supplementary Table S1).

**Figure 3:**
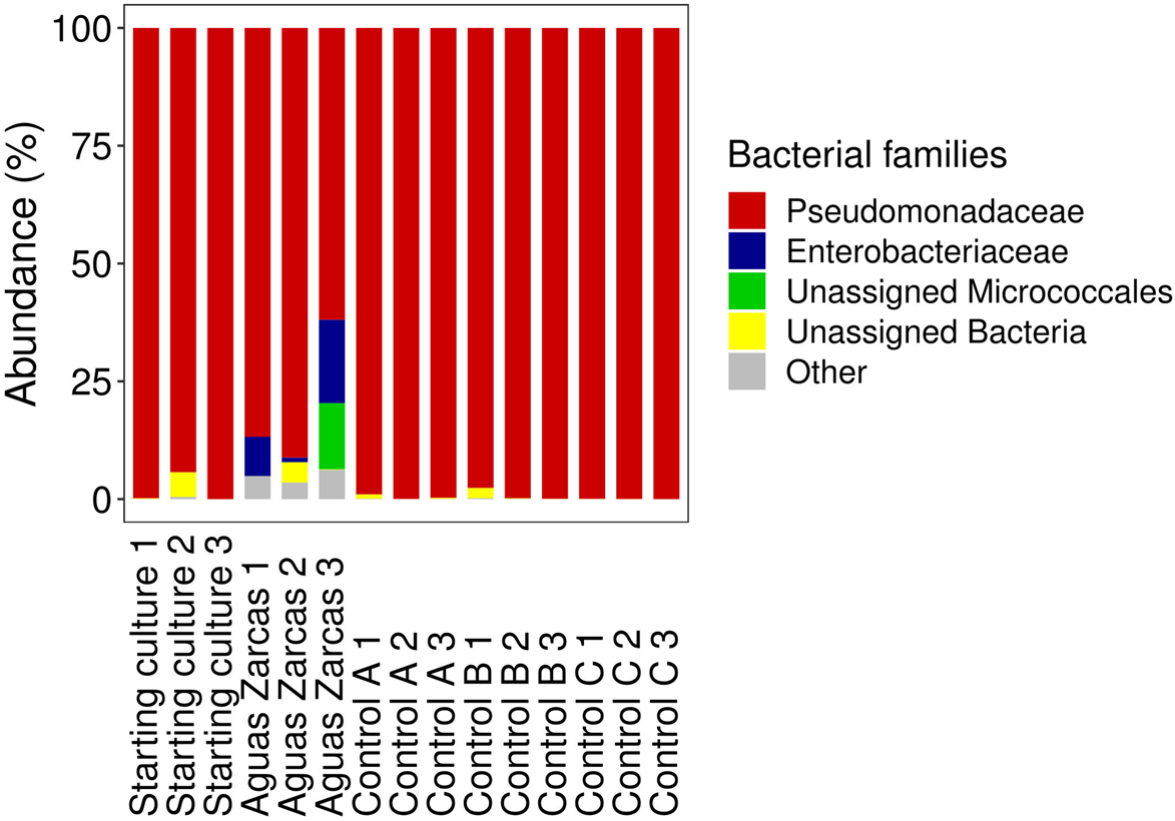
Bacteria growing on the carbonaceous chondrite Aguas Zarcas. Stacked barplot of the abundance of bacterial families present in the starting culture (SC), Aguas Zarcas containing microcosms 14 days after inoculation (Aguas Zarcas), Control A (containing ^13^C-labelled sodium acetate), Control B (non-labelled sodium acetate) and Control C (containing no carbon source). Samples 1, 2 and 3 are biological replicates.

The bacterial community composition is shown in Fig. 3. The starting culture mainly contained Pseudomonadaceae (94–100%), as well as other families at low abundance (e.g. Caulobacteraceae, Carnobacteriaceae, Corynebacteriaceae and Moraxellaceae). After 14 days of growth on Aguas Zarcas, the community composition had shifted significantly. Next to the mainly abundant Pseudomonadaceae (62–91%), a variety of less abundant families was present (e.g. Bacillaceae, Beijerinckiaceae, Burkholderiaceae, Carnobacteriaceae, Clostridiales Family XI, Enterobacteriaceae and Micrococcaceae). Control A, B and C all mainly contained Pseudomonadaceae (98–100%), as well as a few other families at low abundance (e.g. Burkholderiaceae, Micrococcaceae, Sphingomonadaceae).

The pH of the microcosms was measured before inoculation and 14 days after inoculation (“before” and “after” respectively in Supplementary Table S2). A significant difference between the groups was found (ANOVA: *F*-statistic (15,32) = 29.78; *p*-value < 0.001). A *post-hoc* Tukey test showed that the pH of the microcosms containing Aguas Zarcas – both at the start of the experiment and 14 days after incubation—was significantly higher than in all other conditions (*p*-values < 0.001). The pH of Control C significantly increased throughout the experiment (*p*-value = 0.0041), while the pH of none of the other samples significantly changed throughout the experiment.

## Discussion

On early Earth, substantial organic material was being delivered to the surface of the planet in meteoritic material^1,3,5^. Questions persist as to whether this organic material could have allowed a heterotrophic origin of life, and whether after the origin of life, this extraterrestrial material could be a source of organic molecules to power an early heterotrophic biosphere on Earth as well as on other young planets.

This research has shown that bacteria can use organic carbon from the Aguas Zarcas carbonaceous chondrite for cell growth, investigated by combining reverse stable isotope labelling with infrared spectroscopy. This was done by focusing on the incorporation of ^12^C or ^13^C into bacterial biomass as evidenced by vibration shifts in the carbonyl (C=O) stretches in amide I from proteins. The results showed that the amide I peak of ^13^C-labelled bacteria (from growth on ^13^C-labelled sodium acetate as the sole carbon source) shifted the ^13^C amide I peak at 1616 cm^−1^ in the starting culture to the ^12^C amide I peak at 1657 cm^−1^ after growth on Aguas Zarcas, demonstrating the incorporation of carbon (from ^12^C substrates) from the meteorite into the bacteria. The same shift was observed in control bacteria after growth on ^12^C-sodium acetate (Control B). On the other hand, after growth on ^13^C-labelled sodium acetate (Control A), and in the control in which the bacteria were transferred to conditions without a carbon source (Control C), the bacteria retained their ^13^C labelling, showing a ^13^C amide I peak at 1616 cm^−1^. The latter result shows that the ^13^C amide I peak is not lost during starvation, nor that there was any ^12^C-carbon contamination in the experimental setup that could have caused the shift to 1657 cm^−1^ as observed in the Aguas Zarcas samples. These results show that the sole ^12^C amide I peak in the Aguas Zarcas containing samples originates from the meteoritically-associated carbon as the carbon source.

Carbonaceous chondrites on early Earth could thus have provided microorganisms with a substrate, as well as biologically available organics such as nucleobases, amino acids and polycyclic aromatic hydrocarbons^9–12,23^. The biologically available organics in carbonaceous chondrites would locally provide concentrated solutions that could be used by early life^15^. An increase in biodiversity by the presence of extraterrestrial carbon and nutrient sources could have occurred, as well as favouring the growth of heterotrophs or iron and sulphur reducers^24^.

The microbial community composition after growth on Aguas Zarcas mainly consisted of Pseudomonadaceae, as in the starting culture. Some Pseudomonadaceae species are facultative anaerobes, for example the *Pseudomonas* and *Rhizobacter* genus, which can use nitrate as an alternative electron acceptor^25^. Carbonaceous chondrites including Aguas Zarcas contain water-soluble nitrate, albeit in low amounts^15,26,27^. Furthermore, *Pseudomonas* species can degrade organic recalcitrant compounds^25^, which are highly abundant in carbonaceous chondrites. Although microorganisms on early Earth were likely to be different from present-day microorganisms, similarities may exist. For example, *Pseudomonas* can anaerobically perform arginine deiminase, a pathway that is thought to be a primitive remnant from early life on Earth^25,28^.

Next to Pseudomonadaceae, Aguas Zarcas also supported the growth of bacterial families that were not observed in the starting culture or the controls (e.g. Bacillaceae, Beijerinckiaceae, Clostridiales Family XI, Enterobacteriaceae and Steroidobacteraceae). However, these families are assumed to also have been present in very low concentrations in the starting culture. Due to the low biomass in the microcosms, the sequence depth was 5,463 reads. This could mean that families with a low abundance in the inoculum could have been undetected using the 16S rRNA sequencing analysis. The rare biosphere is often underrepresented due to detection limitations, but microbial communities are known to be skewed towards a few dominant species and a high number of rare species^29–31^. Although some of these families could potentially be contaminants, the fact that these families were not observed in any of the controls limits the chance of laboratory contamination as the source of these families. In addition, no bacterial contamination was observed on Aguas Zarcas, which was tested by incubation of the meteoritic powder and by colony-forming unit (CFU) counting (data not shown).

Although microbial incorporation of carbon from Aguas Zarcas was shown, the microbial community grew slower than in the control experiments. A potential reason for this is that the pH of Aguas Zarcas containing microcosms was higher than the other microcosms, including the starting culture. Another explanation could be that Aguas Zarcas created different, and potentially less favourable, geochemical and ionic environmental conditions in the microcosms compared to the starting culture and the controls, for example by the leaching of water-soluble material or ions from the silicate matrix.

Although Aguas Zarcas is considered to be one of the most pristine carbonaceous chondrites, recently, an analysis of the meteorite fragments found terrestrial organic contaminant molecules^32^. This is usually the case for all material that makes contact with the Earth’s atmosphere and surface. Although terrestrial organic contamination of Aguas Zarcas was observed^32^, the total number of detected organics by Tunney et al. was not as extensive as in other research^8,33^. For example, only two amino acids were discovered in one of the five specimens, while previously a wide range of amino acids was detected in Aguas Zarcas^34^. In addition, the organic content of Aguas Zarcas shows bulk similarities with the Murchison CM2 chondrite^7,25^, for example in the aliphatic amines and monocarboxylic acids^8,26^.

The contaminants observed by Tunney et al. were grouped into five categories: agricultural products, fuels, pesticides, pharmaceuticals and plastics^32^. Many of these compounds are not easily degradable^35,36^. Therefore, it is unlikely that the microorganisms in the present study will have avoided meteoritic carbon and preferentially consumed the terrestrial contaminants.

Unfortunately, any meteorite that has landed on Earth is subject to some potential contamination. The material used in this study is considered relatively pristine. However, in order to eliminate terrestrial contamination completely, we suggest the repetition of this experiment with freshly collected material returned from carbonaceous asteroids, for example, material obtained from the asteroid Bennu in the NASA OSIRIS-REx sample-return mission.

The results of this research show that the theory that meteoritic organics are too heterogeneous for microbial use^19^ seems unlikely since we have shown that heterotrophy driven by extraterrestrial organic carbon can be a viable mode of energy acquisition on early Earth. Like the early Earth, the habitability of other young, terrestrial planets could also benefit from the influx of external organics^5^. These beneficial results would be even more pronounced on planets with limited biologically available organics on the surface, and the influx of meteoritic material could thus significantly increase the habitability of a planet. Further, these results indicate that the universe is a potentially vast store of accessible organic carbon to drive heterotrophy. The results also show that organic carbon produced in protoplanetary discs could power an early biosphere on other young planets. The recent discovery of molecular precursors to complex organic materials by the James Webb Space Telescope (JWST) shows that the production of organic molecules is widespread in early planet-forming regions^37^.

Finally, our results show that anaerobic microbial communities can be used to access and metabolically transform carbonaceous asteroidal material in future human space settlement schemes. Carbon-rich extraterrestrial resources could potentially be used as feedstock in anaerobic bioreactors for the production of end products such as rocket propellant and plastics to support a sustainable human presence in space. Here, we have demonstrated the proof-of-concept that organic material and the associated silicate matrix in carbonaceous chondrites are not toxic to anaerobic organisms and are metabolically accessible to power growth, which is the first step in such schemes. Synthetic biology might be used to engineer organisms using the pathways we have demonstrated here to carry out these useful metabolic transformations.

## Methods

### Microbial community

An anoxic, environmental microbial community was sampled from pond sediment of Blackford Pond, Edinburgh, UK according to Waajen et al.^16^. Pond sediment was transported to the laboratory, where the microorganisms were grown anaerobically in microcosms containing the CM2 carbonaceous chondrite Cold Bokkeveld according to Waajen et al.^16^. After three transfers over the course of six months, the stable anaerobic community was stored in 25% glycerol at − 80 °C for further use.

### Meteoritic material

After the initial establishment of a stable community by the CM2 carbonaceous chondrite Cold Bokkeveld, the CM2 carbonaceous chondrite Aguas Zarcas was used for all further experiments. Aguas Zarcas was an observed fall of a highly brecciated^38^, clay- and organic-rich^39^ CM2 carbonaceous chondrite in 2019 in Costa Rica. The used specimen has been collected pre-rain, thereby minimizing terrestrial contamination and weathering^39^. Due to the short terrestrial residence time, Aguas Zarcas is one of the most pristine extraterrestrial materials on Earth. Aguas Zarcas has low terrestrial organic contamination^26^, but like any meteorite that has been in contact with Earth’s surface, it is not completely free from terrestrial organics^32^. Aguas Zarcas is rich in hydrocarbons, carboxylic acids, dicarboxylic acids and macromolecular organics, and is similar to the Murchison CM2 chondrite^8^; but contains relatively low levels of ammonia, amino acids and amines^8,26^.

The carbon content of the meteorite was analysed using an Elemental Analyser—Isotope Ratio Mass Spectrometry (EA-IRMS). Samples of ≤ 40 mg powdered meteorite in tin capsules were loaded into an auto-sampler on a Europa Scientific elemental analyser. Samples were dropped into a furnace in an oxygen-rich environment, and heated from 1000 to ~ 1700 °C. A helium stream transported produced gases over combustion catalyst wires with chromium trioxide and copper oxide to oxidise hydrocarbons and silver wool to remove sulphur and halides. The resulting gases N_2_, NO_x_, H_2_O, O_2_ and CO_2_ were passed through a reduction stage of pure copper wires at 600 °C to remove O_2_ and convert NO_x_ species to N_2_. Water was removed using a magnesium perchlorate chemical trap. A packed column gas chromatograph at 100 °C separated CO_2_ from N_2_. The resulting CO_2_ chromatographic peak was then ionised and accelerated by the ion source of the Europa Scientific 20–20 IRMS. A magnetic field separated gas species of different mass, while the isotopomers of CO_2_ at *m*/*z* 44, 45 and 46 were measured using a Faraday cup collector array.

The reference material used was IA-R001 (wheat flour, δ^13^C_V-PDB_ = − 26.43 ‰). IA-R001 was used in combination with IA-R005 (beet sugar, δ^13^C_V-PDB_ = − 26.03 ‰) and IA-R006 (cane sugar, δ^13^C_V-PDB_ = − 11.64 ‰) for quality control purposes. IA-R001, IA-R005 and IA-R006 were calibrated against the inter-laboratory comparison standard IAEA-CH-6 (sucrose, δ^13^C_V-PDB_ = − 10.43 ‰), which was distributed by the International Atomic Energy Agency (IAEA), Vienna.

### Preparation of anaerobic cultures

Microcosms were prepared in 12.5 mL glass serum bottles with butyl rubber stoppers. Glassware was made organic-free as described in Waajen et al.^16^ following Eaton et al.^40^. Butyl rubber stoppers were washed with common detergent and copiously rinsed with distilled water. Then, the stoppers were boiled three times for 5 min in Invitrogen™ UltraPure™ DNase/RNase-Free Distilled Water and air-dried.

Microcosms were made containing 5 mL M9 medium (3 g/L KH_2_PO_4_, 7 g/L Na_2_HPO_4_, 1 g/L NH_4_Cl, 0.5 g/L NaCl, 0.12 g/L MgSO_4_, 0.011 g/L CaCl_2_) with 3.47 g/L of either regular CH_3_COONa or double ^13^C-labelled CH_3_COONa (double labelled being that both ^12^C atoms were replaced with ^13^C atoms in the molecule; also denoted as [^13^C_2_]-CH_3_COONa) as the sole carbon source and sterile Invitrogen™ UltraPure™ DNase/RNase-Free Distilled Water. In addition, microcosms were prepared containing 0.5 g powdered Aguas Zarcas or Cold Bokkeveld in 5 mL sterile Invitrogen™ UltraPure™ DNase/RNase-Free Distilled Water. The meteorites were handled under sterile conditions, broken into pieces with a heat-sterilised chisel and powdered with an organic-free mortar and pestle. These had been sterilised by heating to 550 °C for 6 h in a Carbolite 1100 °C Chamber Furnace. Microcosms were made anoxic by flushing with sterile N_2_ gas.

### Microbial growth

The cryostock of the stable anaerobic community was used as the starting culture for the experiment. Three anoxic microcosms containing 5 mL M9 with [^13^C_2_]-CH_3_COONa were inoculated with 20 μL of the cryostock. These microcosms were incubated for approximately two weeks, shaking at 150 rpm at room temperature (20–25 °C) after which they were transferred to fresh microcosms with [^13^C_2_]-CH_3_COONa. This process was repeated twice, after which the community was transferred to the following anoxic microcosms in triplicate: (1) Containing 0.5 g powdered Aguas Zarcas and 5 mL sterile Invitrogen™ UltraPure™ DNase/RNase-Free Distilled Water; (2) Containing 5 mL M9 medium with 3.47 g/L [^13^C_2_]-CH_3_COONa (Control A); (3) Containing 5 mL M9 medium with 3.47 g/L non-labelled CH_3_COONa (Control B); (4) Containing 5 mL sterile Invitrogen™ UltraPure™ DNase/RNase-Free Distilled Water (Control C).

These communities were incubated for 14 days, except for the communities on powdered Aguas Zarcas (1), which were incubated for 33 days due to their slower growth rate. Non-biological controls of each of these conditions were also performed, where the four conditions above were incubated in the absence of the microbial community.

Microbial growth was tested by colony-forming unit (CFU) counts on anoxic LB agar plates (adapted from DSM 381 (DSMZ; Deutsche Sammlung von Mikroorganismen und Zellkulturen): 10.0 g/L tryptone; 5.0 g/L yeast extract; 5.0 g/L NaCl; 20 g/L agar; pH 7.0) at room temperature. Agar plates were plated by single plate-serial dilution spotting (SP-SDS)^41^. SP-SDS of the communities in microcosms were conducted both immediately after incubation and 14 days after incubation. The communities on Aguas Zarcas were also spotted with SP-SDS 33 days after inoculation.

All experiments were carried out in triplicate, except for the non-biological control with Aguas Zarcas, which was conducted once due to limitations in the amount of material. Prior pilot experiments in triplicate showed no contamination of microbial growth on Aguas Zarcas (data not shown).

### pH

The pH of the microcosms was measured prior to inoculation and 14 days after inoculation using a Jenway 3510 pH meter (Cole-Parmer, Staffordshire, UK) and InLab Semi-Micro-L pH electrode (Mettler Toledo Ltd, Leicester, UK).

An ANOVA and post-hoc Tukey test were performed to compare the pH of the microcosms prior to inoculation to 14 days after inoculation; the pH of the different conditions prior to inoculation to each other; and the pH of the different conditions 14 days after inoculation to each other.

### O-PTIR and FT-IR

After the incubation period, anoxic aliquots were shipped to the University of Liverpool, where biological samples were prepared for optical photothermal infrared (O-PTIR) spectroscopy according to Lima et al*.*^22^. Samples were washed three times by repeatedly centrifuging the cells for 10 min at 5000×*g* at room temperature using a benchtop Eppendorf microcentrifuge 5424R (Eppendorf Ltd., Cambridge, U.K.), discarding the supernatant and resuspending the samples in 2 mL deionised water. Five microliters of each cell suspension were spotted on CaF_2_ substrates and left to dry in a desiccator at room temperature.

A mIRage infrared microscope (Photothermal Spectroscopy Corp., Santa Barbara, USA) with a tunable four-stage QCL device and a continuous wave 532 nm laser probe beam was used to acquire O-PTIR measurements using the single-point and imaging mode. Using reflection mode, spectral data were collected using a 40 ×, 0.78 NA, and 8 mm working distance Schwarzschild objective. A spectral region of 800–1800 cm^−1^ was analysed with 2 cm^−1^ spectral resolution and 50 scans per spectrum to obtain single-point spectral data. Single frequency images were collected at a 500 nm step size by tuning the QCL device to the frequencies corresponding to amide I of labelled and unlabelled cells (1616 and 1657 cm^−1^ respectively) in order to find the exact location of bacterial cells. PTIR Studio software provided by the manufacturers was used for instrument control and data collection.

Non-biological samples were analysed using Fourier transform infrared (FT-IR) spectroscopy. Twenty microliters of non-biological samples were transferred to 96-well silicon substrates (Bruker Ltd, Coventry, UK) and dried prior to data collection. FT-IR data were acquired in the mid-IR range (4000–600 cm^−1^), with 64 spectral co-adds and using an FT-IR spectrometer (Bruker Invenio, Bruker Ltd, Coventry, UK).

All spectra were pre-processed by baseline correction, smoothed via a Savitzky–Golay filter using a polynomial of second order in an 11-point window (approximately 22 cm^−1^ in size), vector-normalised and mean-centered. After pre-processing, spectra were cut from 1500 to 1760 cm^−1^ and the data were subjected to principal component analysis (PCA) using Quasar version 1.7.0^42,43^. All collected data were processed using MATLAB software version 2011a (Mathworks Inc., Natwick, USA), and all code used for these analyses is available via GitHub (https://github.com/Biospec/).

### 16S rRNA gene amplicon sequencing and analysis

DNA of biological samples 14 days after incubation was extracted using the DNeasy PowerSoil Pro Kit (QIAGEN GmbH, Germany). Additionally, a negative control containing no community sample was examined. Extracted DNA concentration was measured using a Qubit 3 Fluorometer (Life Technologies). Extracted DNA was stored at − 20 °C and shipped on dry ice to the Research and Testing Laboratories (RTLGenomics, Texas, USA) for amplicon sequencing.

Before DNA amplification, the following samples were concentrated by adding 1.2 × their corresponding volumes of SPRIselect Reagent (BeckmanCoulter, Indianapolis, Indiana), followed by two washes of 80% ethanol, and elution in 15µL of 10 mM Tris–Cl, pH 8.5: Control C 3, Aguas Zarcas 1 and Aguas Zarcas 3.

The 16S rRNA gene was amplified with primers 341F (CCTACGGGNGGCWGCAG) and 805R (GACTACHVGGGTATCTAATCC) added to the overhang adapters TCGTCGGCAGCGTCAGATGTGTATAAGAGACAG and GTCTCGTGGGCTCGGAGATGTGTATAAGAGACAG respectively. Twenty-five-millilitre reactions with Qiagen HotStar Taq master mix (Qiagen Inc, Valencia, California), 1 µL of 5 µM of each primer, and 1 µL of template DNA were used in ABI Veriti thermocyclers (Applied Biosystems, Carlsbad, California, USA) for PCA amplification with the following thermal profile: 95 °C for 5 min, then 35 cycles of 94 °C for 30 s, 54 °C for 40 s and 72 °C for 1 min, followed by 1 cycle of 72 °C for 10 min, and hold at 4 °C. Barcodes were added to the sample in a second PCR using first-stage amplicons based on their concentrations, and Illumina Nextera PCR primers (Forward AATGATACGGCGACCACCGAGATCTACAC[i5index]TCGTCGGCAGCGTC and Reverse CAAGCAGAAGACGGCATACGAGAT[i7index]GTCTCGTGGGCTCGG). A thermal profile as follows was used: 95 °C for 5 min, then 10 cycles of 94 °C for 30 s, 54 °C for 40 s and 72 °C for 1 min, followed by 1 cycle of 72 °C for 10 min, and hold at 4 °C. Amplification products were visualised with eGels (Life Technologies, Grand Island, New York, USA). After pooling the products to equal molar concentrations, size selection was performed in two rounds using SPRIselect Reagent (BeckmanCoulter, Indianapolis, Indiana, USA) in a 0.75 ratio for each round. Selected pool concentration was measured using a Qubit 4 Fluorometer (Life Technologies). Libraries were sequenced using Illumina MiSeq (Illumina Inc., San Diego, California, USA) 2 × 300 flow cell at 10 pM.

FASTQ files from RTLGenomics were analysed using the EDGE Bioinformatics Web-based platform which uses automated scripts of QIIME2 version 2019.10.0^44,45^. Operational taxonomic units (OTUs) were used as a quality control method, with a binned OTU representing a sequence similarity of 97%. Sequences with a Q-score of 20 or above were retained. The minimum fraction of consecutive high-quality base calls to include a single end read of the input read length was 0.75. Per sequence, one ambiguous nucleotide was allowed. Sequences were demultiplexed and joined using QIIME2 vsearch commands and methods^46^. The retained sequences were clustered into OTUs with the SILVA-132-99 database and the qiime vsearch cluster-features-open-reference command. UCHIME (qiime vsearch uchime-ref command) was used to remove chimeric sequences. OTUs were taxonomically identified using the q2-feature-classifier plugin^47^. The raw OTU feature table with 1,801 OTUs (qiime phylogeny align-totree-mafft-fasttree command) was used to create a rooted phylogenetic tree by using MAFFT alignment tool^48^ and FastTree method^49^. A community composition barplot was constructed from the rarefied OTU table with a sampling depth of 5,463 sequences (1156 OTUs) after excluding families containing less than 10 sequences per environmental condition, and after removing OTUs present in the negative control with at least 10 reads.

### Supplementary Information

Supplementary Information.

## Data Availability

The datasets generated and/or analysed during the current study are available in the GitHub repository, https://github.com/ACWaajen/AguasZarcas
