# Supplementary material for: Life on Earth can grow on extraterrestrial organic carbon
Source: Sci Rep. 2024 Feb 14;14:3691. doi: 10.1038/s41598-024-54195-6 (PMC10866878; doi:10.1038/s41598-024-54195-6)
Supplement: Supplementary file 1 — Supplementary Information. [file 41598_2024_54195_MOESM1_ESM.docx]

# Supplementary material

| **Sample** | **Time after**  **inoculation (days)** | **Cell concentration (CFU/mL)** |
| --- | --- | --- |
| Aguas Zarcas | 0 | 3.27 x 10^4^ + 2.91 x 10^3^ |
| Aguas Zarcas | 14 | 2.18 x 10^4^ + 1.04 x 10^4^ |
| Aguas Zarcas | 33 | 2.92 x 10^5^ + 2.84 x 10^5^ |
| Control A | 0 | 2.90 x 10^4^ + 2.36 x 10^3^ |
| Control A | 14 | 3.38 x 10^6^ + 1.92 x 10^5^ |
| Control B | 0 | 3.60 x 10^4^ + 3.18 x 10^3^ |
| Control B | 14 | 4.22 x 10^6^ + 8.38 x 10^5^ |
| Control C | 0 | 3.35 x 10^4^ + 2.29 x 10^3^ |
| Control C | 14 | 2.33 x 10^6^ + 2.19 x 10^5^ |

**Supplementary Table 1: Microbial growth on the carbonaceous chondrite Aguas Zarcas.** Colony-forming unit (CFU) counts in microcosms containing Aguas Zarcas, Control A (containing double ^13^C-labelled sodium acetate), Control B (non-labelled sodium acetate) and Control C (containing no carbon source) throughout the experiment. Values are means with standard deviations shown from three replicates.

| **Sample** | **pH** |
| --- | --- |
| Aguas Zarcas before | 8.38 + 0.19 |
| Aguas Zarcas after | 8.15 + 0.34 |
| Control A before | 7.09 + 0.01 |
| Control A after | 7.04 + 0.15 |
| Control B before | 7.09 + 0.02 |
| Control B after | 7.01 + 0.20 |
| Control C before | 6.73 + 0.23 |
| Control C after | 7.30 + 0.17 |
| Starting culture before | 7.09 + 0.01 |
| Starting culture after | 7.11 + 0.01 |
| Non-biological Aguas Zarcas before | 8.55 |
| Non-biological Aguas Zarcas after | 8.34 |
| Non-biological Control A before | 7.11 + 0.01 |
| Non-biological Control A after | 7.10 + 0.02 |
| Non-biological Control B before | 7.11 + 0.01 |
| Non-biological Control B after | 7.11 + 0.04 |
| Non-biological Control C before | 6.78 + 0.24 |
| Non-biological Control C after | 6.51 + 0.07 |

**Supplementary Table 2: pH of microcosms.** pH results of the biological and non-biological microcosms before inoculation and 14 days after inoculation (mean + standard deviation). All conditions were tested in triplicate, except for non-biological Aguas Zarcas, which was tested once.

**Supplementary Figure 1: Influence of carbon source isotopes on bacterial separation in principal component analysis (PCA).** Principal component (PC) loadings contribution from the PCA of the biological samples (Figure 2) for a) PC1 and b) PC2. Vertical dotted lines show the positions of the Amide I bands originating from carbonyl vibrations containing either ^12^C (1657 cm^-1^) or ^13^C (1616 cm^-1^).


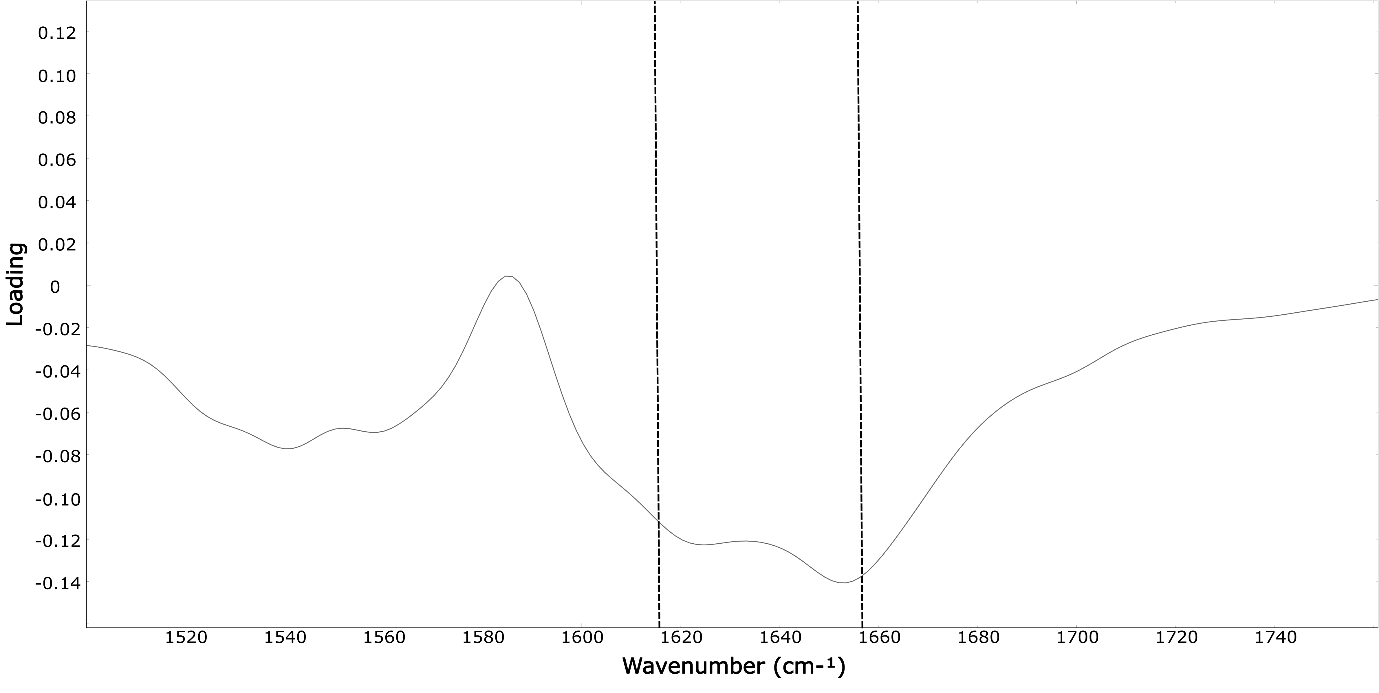

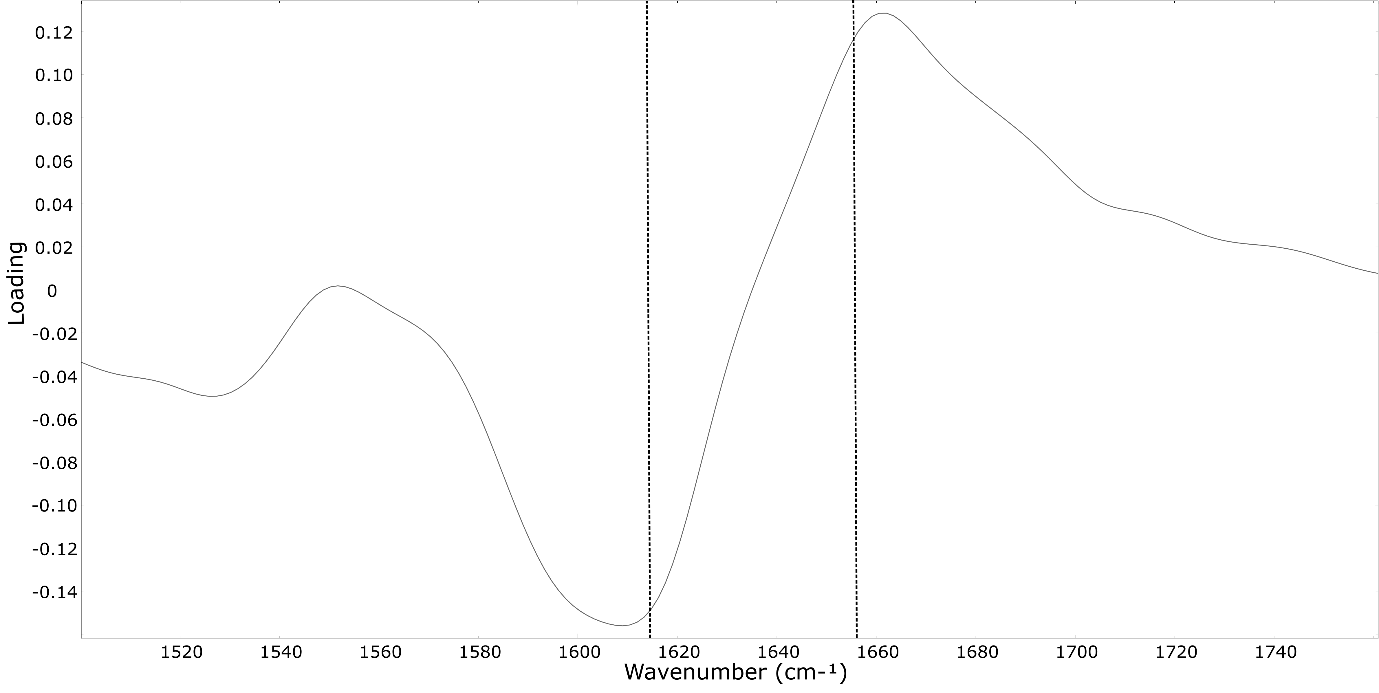


b)

a)

**Supplementary Figure 1: Principal component (PC) loading plots showing the influence of carbon source isotopes on bacterial separation in principal component analysis (PCA).** Principal component (PC) loadings contribution from the PCA of the biological samples (figure 2) for a) PC1 and b) PC2. Vertical dotted lines show the positions of the amide I bands originating from the carbonyl vibrations containing either ^12^C (1657 cm^-1^) or ^13^C (1616 cm^-1^).


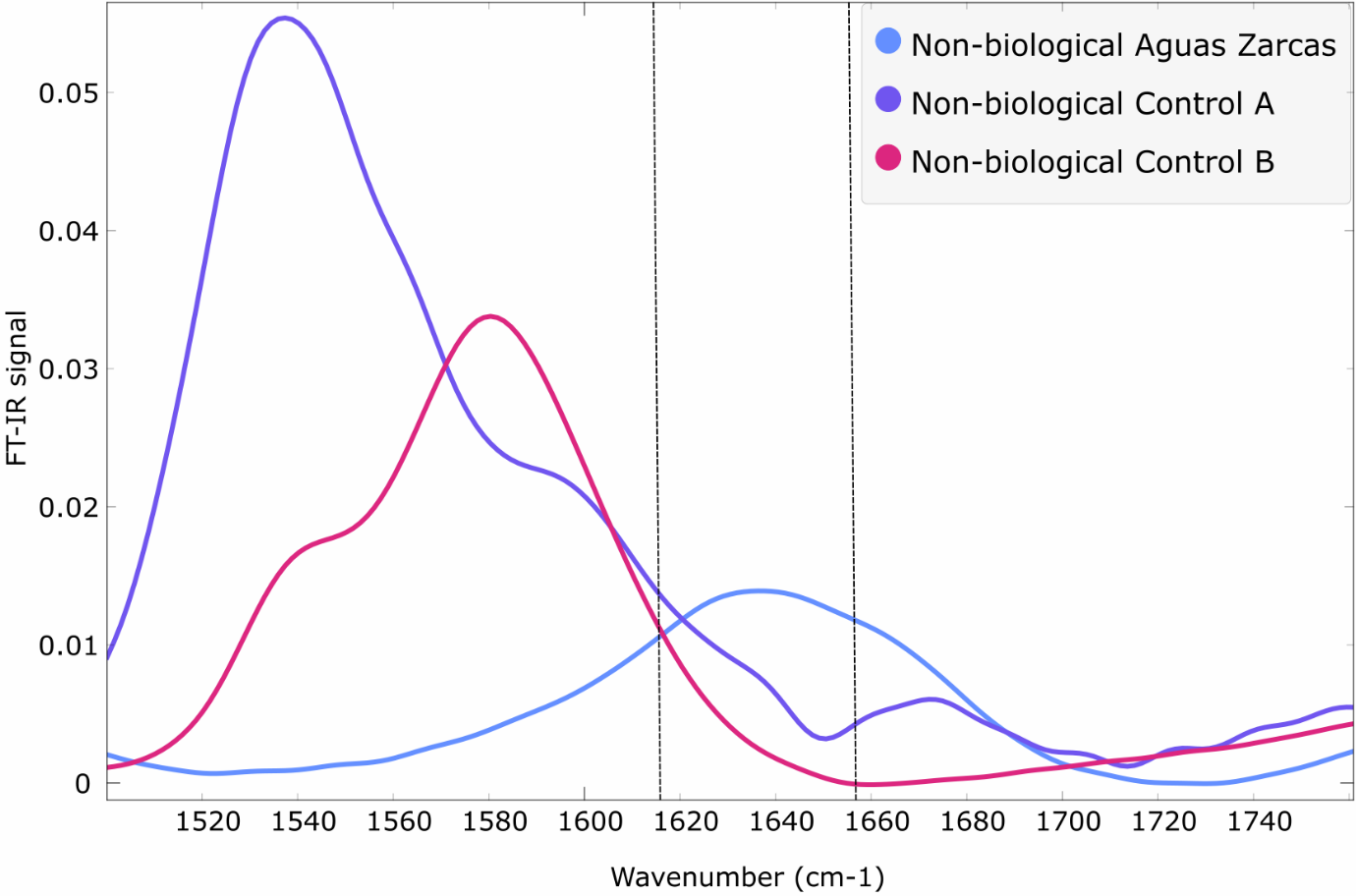


**Supplementary Figure 2: Growth medium and Aguas Zarcas meteorite do not contribute to amide I peaks**. Fourier transform infrared (FT-IR) spectroscopy results of non-biological samples from 1500 to 1760 cm^-1^. No peaks are observed at the amide I peaks for ^12^C (1657 cm^-1^) and ^13^C (1616 cm^-1^), indicated by vertical dotted lines.
